# Vascular Endothelial Growth Factor Receptor, fms-Like Tyrosine Kinase-1 (Flt-1), as a Novel Binding Partner for SARS-CoV-2 Spike Receptor-Binding Domain

**DOI:** 10.3389/fimmu.2022.906063

**Published:** 2022-07-08

**Authors:** Adel Zaid I. Mutahar, Manjunath Devaramani, Renu Dayal, Dinesh Kumar Saini, Paramahans V. Salimath, Bharathi P. Salimath

**Affiliations:** ^1^ Department of Studies in Biotechnology, University of Mysore, Mysore, India; ^2^ Medical Laboratories Department, Abs Community College, Hajah, Yemen; ^3^ Denovo Biolabs Pvt. Ltd., Bangalore, India; ^4^ Department of Biotechnology, Sanorva Biotech Pvt. Ltd., Mysore, India

**Keywords:** SARS-CoV-2, recombinant spike RBD, 2-domain soluble Flt-1, binding partners, antibodies

## Abstract

Angiotensin-converting enzyme 2 (ACE2) and neuropilin 1, a vascular endothelial growth factor (VEGF) receptor, were identified to bind to the SARS-CoV-2 spike receptor-binding domain (spike RBD). *In silico* analysis based on 3D structure, multiple sequence alignment, and molecular docking of second domain of soluble Flt-1 (sFlt-1) and spike RBD revealed structural similarities, sequence homology, and protein-protein interaction. Interaction and binding of recombinant spike RBD (rspike RBD) and recombinant sFlt-1 (rsFlt-1) *in vitro* induced a conformational change, as revealed by spectrofluorimetric data, with increased fluorescence intensity in emission spectra as compared to either of the proteins alone. Results on ELISA confirmed the binding and cross-reactivity of rspike-RBD and rsFlt-1 as determined by using either specific antibodies towards each protein or immunized human serum. We found that polyclonal or monoclonal anti-spike RBD antibodies can recognize either rsFlt-1 or rspike RBD, showing cross-reactivity for the two proteins in a dose-dependent binding response. Recognition of bound rspike RBD or rsFlt-1 by anti-Flt-1 or anti-spike RBD antibodies, respectively, as observed by immunoblotting, further confirmed interaction between the two proteins. Immunoprecipitation and immunoblot analysis demonstrated the identification of rspike RBD binding to the Flt-1 receptor on A549 cells. Further, the binding of rspike RBD to Flt-1 receptor was shown using immunofluorescence on 2D-culture or 3D-spheroid of MDA-MB-231 cells, which over-express Flt-1 receptor. Together, our study concludes that the Flt-1 receptor is a novel binding partner for SARS-CoV-2 spike RBD.

## Introduction

The continuing outbreak of Coronavirus Disease 2019 (COVID-19), caused by the severe acute respiratory syndrome coronavirus 2 (SARS-CoV-2), has created a significant and urgent need to identify and develop safe, effective new therapies against virus causative agents (1, 2). Several studies on the process of SARS-CoV-2 viral attachment and invasion of human cells reported viral entry by different cellular receptors. These studies confirmed that the S protein of SARS-CoV-2 uses ACE2 as a host surface receptor to enable viral entry and infect the cell (3, 4). The C-terminal domain of S-protein, which is also known as the receptor-binding domain (RBD), is known to be responsible for binding to ACE2 (5). Additionally, neutralizing antibodies bind to S-protein, and any mutations in S-protein RBD could lead to immune escape, which is detrimental to current therapies and vaccines (6). In addition to ACE2, CD147 protein has been recognized as a co-receptor in host cells to augment the ability of SARS-CoV-2 to enter human cells (7–9). Enzymatic activation of the S1 and S2 subunits of the spike protein needs to be cleaved by host proteases. As a co-expressed membrane endopeptidase of the ACE2 receptor, Furin can cleave the viral envelop S1 and S2 glycoprotein units, allowing for successful viral fusion with host cell membranes (10). Besides ACE2 and CD147, Neuropilin-1 (NRP-1, a VEGF receptor) was recently identified as a novel receptor for SARS-CoV-2 entry to the host cell (11). Pulmonary vascular endothelialitis is the main feature of the most severely affected COVID-19 patients, which leads to respiratory failure, thrombosis, and multi-organ dysfunction (12).

As a consequence of the binding of SARS-CoV-2 to ACE2, elevated levels of VEGF and angiotensin II (Ang-II) promote vascular permeability and inflammation, leading to acute lung injury (ALI) (13). Similarly, vascular endothelial growth factor receptor1 (VEGFR1/sFlt-1) is highly expressed on epithelial cells and shows a strong binding affinity for VEGF. Intussusceptive angiogenesis has been reported in the lungs of patients who died from respiratory failure caused by COVID-19 compared to patients who died from influenza (14). Therefore, VEGF-targeted medicine and VEGF receptors may provide a novel strategy for treating ALI/ARDS induced by COVID-19 infection (15). Dupont et al. (16) reported a link between sFlt-1 and an endothelial dysfunction biomarker, soluble vascular cell adhesion molecule-1, in COVID-19 patients with high circulating levels of sFlt-1. We have undertaken these studies because of the lack of studies on the interaction of spike RBD and Flt-1, and subsequent manifestations of the interactions are less well understood.

Furthermore, *in vitro* cell-based assays were also used to validate the Flt-1 and spike RBD interactions. Herein, our study reports for the first time that Flt-1 is a novel binding receptor for spike-RBD of SARS-CoV-2. Therefore, we aimed to understand the interaction between spike-RBD and Flt-1 proteins and their respective antibodies, which may be utilized in the controlling and treating pathogenesis of COVID-19 patients.

## Materials and Methods

### Materials

Adenocarcinoma human alveolar basal lung epithelial cells (A549 cells) and MDA-MB 231 cells were purchased from the National Centre for Cell Science (NCCS, Pune, India). Dulbecco’s Modified Eagle’s Medium (DMEM), Iscove’s Modified Dulbecco’s Media (IMDM), and all other chemicals required for cell culture were purchased from Gibco, Invitrogen, USA. VEGF, rabbit polyclonal anti-VEGF antibody, and rabbit polyclonal anti-sFlt-1 antibody (Sanorva Biotech Pvt. Ltd, India). Rabbit polyclonal anti-spike RBD HRP tagged secondary antibody, mouse monoclonal anti-spike RBD, Goat anti-human IgG- horseradish peroxidase (HRP) conjugated antibody (Denovo Biolabs Pvt. Ltd, India). Goat anti-rabbit IgG HRP tagged (GeNei). Mouse monoclonal p-Tyr was purchased from Santa Cruz Biotechnology, USA. Alexa Fluor 488 and Alexa Flour 594 Phalloidin were purchased from Invitrogen, Molecular Probes, USA. Hoechst 33342 purchased from Thermofisher, USA. Methylcellulose, DAPI, and DAB-H_2_O_2_ were purchased from Sigma-Aldrich. Nitrocellulose membrane (PALL Corporation, USA). Enhanced chemiluminescence ECL western blot substrate reagents (Bio-Rad, USA). All other chemicals and reagents used were of high analytical grade.

### Identification of Homologies Between sFlt-1 and SARS-CoV-2 Spike-RBD Proteins and Comparison of Their 3D Structures

The following protein sequences were obtained from UniProtKB/Swiss-Prot portal: spike-RBD protein (231 aa) - P0DTC2; sFlt-1(94 aa**)-** P17948**, ACE2 (597 aa)-** Q9BYF1**, and neuropilin-1 (166 a.a**.) UniProt O14786. The 3D structure of spike-RBD and sFlt-1 proteins were recovered from the UniProt 3D structure PBD database (PDB DOI: 10.2210/pdb6W41/ PDB And: 10.2210/pdb4CKV/PDB). sFlt-1 and spike-RBD 3D structures were compared by referring to the published structure of the proteins (17, 18). Additionally, sequence alignment of amino acid residues of sFlt-1 with either spike RBD or ACE2 and neuropilin-1 which are known binding partners of spike RBD was performed using Clustal Omega multiple sequence alignment tools.

### *In Silico* Computational Studies

#### Protein-Preparation

Molecular docking studies were performed using Auto Dock 4 version starting by three-dimensional (3D) structure of SARS-CoV-2 spike RBD protein preparation retrieved from protein data bank (PDB)- 6W41 deposited in Research Collaboratory for Structural Bioinformatics (RCSB) (17). The protein structure was prepared by deleting the existing extra chains (Chain A and B in 6W41 3D structure), water molecules removed, and hydrogen atoms and Gasteiger charges were added. Prepared protein was processed for molecular docking studies with domain 2 of the sFlt-1 peptide as a ligand.

#### Ligand Preparation

Ligand was prepared by retrieving d2-sFlt1 3D- structure from protein data bank (PDB)- 4CKV deposited in Research Collaboratory for Structural Bioinformatics (RCSB) (18). Detection and selection of the reaction roots optimized, then choosing and setting the number of active torsions using ligand preparation tools of Auto Dock 4.

#### Computational Docking Studies

SARS-CoV-2 spike RBD protein (6W41) was chosen as a macromolecular and grid box with X, Y, and Z dimensions were set to contain the active site of the RBD protein molecule. The Auto grid program generated affinity (grid) maps of 2 Å grid points and 0.414 Å spacing. AutoGrid4 was run to generate the grid parameters. Docking simulations were performed using the programmed genetic algorithm inbuilt in the Auto Dock 4.2 version. The initial position, orientation, and the number of torsions of the ligand molecules were set randomly. All rotatable torsions were released during docking. Each docking simulation was derived from two runs that were set to terminate after a maximum of 250,000 energy evaluations. The population size was set to 150. During the docking process, a number of torsions were set into 29 torsion atoms. Docking parameters were analyzed using analyze docking results in the Auto Dock 4.2 version showing the interactions between the receptor and ligand with the lowest free energy of binding.

### Recombinant sFlt-1 and Spike RBD Production

Recombinant sFlt-1, with extracellular 2-domains (2D), was cloned and expressed as previously described (19). Stabilized spike (RBD) construct was capped at the C-terminus with a His-tag. Spike (RBD) S1-pET28a clone was synthesized and transformed into Rosetta (DE3). The overnight culture was induced with 1mM IPTG for 4 hours at 16°C. The cell pellet was dissolved in 8M urea prior to purification using Ni-NTA column chromatography.

#### Confirmation of rsFlt-1 and rspike RBD Purification by SDS-PAGE and Western Blot

The purified rsFlt-1 and rspike RBD proteins were analyzed by SDS-PAGE and western blotting. The blot was blocked with 5% skimmed milk in Tris-buffered saline containing 0.5% Tween-20 (TBST) for 2 hours at 37°C and further incubated with either anti-sFlt-1 polyclonal antibody (rabbit anti-sera) or COVID19 positive or negative antisera respectively, overnight at 4°C. After washing with PBST, the blots were probed either with HRP-conjugated goat anti-rabbit IgG or goat anti-human IgG- HRP conjugated antibodies. Post incubation and development using ECL reagent or DAB-H_2_O_2_, respectively, the blots were processed for visualization. for 2 hours at room temperature and then visualized by gel documentation unit (Bio-Rad) using an enhanced chemiluminescence ECL western blot substrate reagents.

### Production of Polyclonal and Monoclonal Antibodies

Polyclonal antibodies against rsFlt-1 and rspike RBD were raised in pathogen-free New Zealand white rabbits by using standard protocols. Rabbit polyclonal IgG was purified by ammonium sulfate precipitation and protein A agarose column chromatography. Monoclonal antibodies of rspike RBD were produced by fusing splenocytes from rspike RBD immunized Balb/c mice with SP2-Ag14 myeloma cells (ATCC) and cultured in IMDM using a standard protocol. Hybridoma supernatant from confluent wells was screened against 2 µg/mL of rspike RBD by indirect ELISA. More than 90 positive monoclonal antibody clones were identified (result shown in **Supplementary Data**).

### Protein-Protein Interaction Based on Fluorescence of Intrinsic Aromatic Amino Acids

Emission spectra of rspike RBD protein, rsFlt1, VEGF protein alone, and a mixture of sFlt-1 -rspike RBD or sFlt-1-rspike RBD-VEGF recombinant proteins were assessed using a fluorescence scan method (spectrofluorometer), in a range of 280 nm to 350 nm (20). Briefly, 100 ng of each recombinant protein in 100μl of PBS was added to the designated wells in a 96-well plate. After 2 hours of incubation at 37°C, fluorescence intensity scanning was recorded using a multimode microplate reader (TECAN infinite 200Pro, Austria). Furthermore, time kinetics of protein-protein interactions were also performed on either free or complex proteins, including negative (free protein alone) or positive control, using rVEGF and rsFlt-1 interactions. The fluorescence emission was measured using a multimode microplate reader. All experiments were performed in triplicate.

### Enzyme-Linked Immunosorbent Assay (ELISA)

To determine the binding and cross-reactivity of rspike RBD to rsFlt-1 and their antibodies, three methods of indirect ELISA were used as follows. Commonly PBST, pH 7.4 containing 0.05% Tween-20 was used as washing buffer. Post-immobilization, overnight incubation was done at 4C, skimmed milk (5%) was used as blocking buffer in all three methods. To assess the binding of rspike RBD to rsFlt-1, different concentrations of rspike RBD (0.1, 0.25, 0.5, and 1 µg) were immobilized on an ELISA plate. Likewise for assessing binding of rsFlt-1 and rspike RBD, different concentrations of rsFlt-1 (0.1, 0.25, 0.5, 1 µg and/or 0.2, 0.5, 1, and 2.5 µg) were immobilized on ELISA plate. After overnight incubation, plates were washed with washing buffer. Either different doses of rsFlt-1 (0.01, 0.1, and 1 µg) or rspike RBD (at 1 µg or 0.01, 0.1, 1 µg) were added to either rspike RBD or rsFlt-1 immobilized wells, respectively. ELISA plate was blocked for 1 hour and washed prior to adding rabbit polyclonal anti-sFlt-1 antibody (1:500) to spike RBD immobilized wells. Goat-anti-rabbit ALP-tagged secondary antibody at 1:1000 dilution was used for rspike coated wells. For the wells immobilized with sFlt-1, either rabbit-polyclonal HRP tagged anti-spike RBD (1:750), or anti-spike monoclonal antibody (clone 7E3) was used as primary antibody. To the designated wells where monoclonal antibody was used, goat-anti-mouse HRP conjugate was used as a secondary antibody (1:8000). After washing, the plates were developed either with PNPP or TMB/H_2_O_2_, and absorbance was measured at 405 nm or 450 nm. The data was analyzed and represented using GraphPad prism software.

### Binding of rspike RBD With Antibodies Present in Vaccinated Serum

To verify and delineate the mechanism of neutralizing viral infection in vaccinated individuals, serum from vaccinated (2 months post-COVID19 vaccination) and non-vaccinated individuals (healthy volunteers) were used to perform indirect ELISA. Non-vaccinated serum and nucleocapsid-protein (N-protein) were used as controls. In brief, 96-well microtiter plate (Nunc™ Microtiter Well plates) was coated with either rspike RBD (0.1µg) or rsFlt-1 (0.3µg) or N-protein (0.1µg) or rspike RBD: sFlt-1 protein (in 1:3 ratio) in coating buffer (0.5M sodium bicarbonate, pH 9.6). After overnight incubation at 4°C, wells were washed 5 times with 1X PBST, followed by blocking with 5% skimmed milk for 2 hours. After washing, vaccinated and non-vaccinated serum samples pre-diluted with PBS in a ratio of 1:10, 1: 100, 1: 1000 and 1:10000 were added to the respective wells and incubated overnight at 4°C. The plate was washed with PBST, then goat-anti-human HRP-tagged secondary antibody was added, and the plate was incubated at 37°C for two hours. The reaction was developed using PNPP and read at 405 nm. The data was analyzed and represented using GraphPad prism software.

### Cytotoxicity Assay

A549 cells (5 x 10^4^) were seeded in 1 ml of complete media (DMEM supplemented with fetal bovine serum (10%) and penicillin-streptomycin antibiotic (1%)) in 24-well plates and treated with 1, 5, 10, 50, and 100ng of rspike RBD or rsFlt-1 for 48 hours and the cells were trypsinized, stained with trypan blue for 2 minutes. The cells were counted using a hemocytometer. For counting, 10μl of cells was taken along with 10μl of trypan blue dye (0.4% in 980μl of PBS) in a micro-centrifuge tube and allowed to stand for 3 minutes. The cell concentration per mL was calculated using the formula: (Average cell count x dilution factor x 10^4^). The percentage of viable cells was normalized to untreated cells, and the cell viability was calculated and represented using GraphPad prism 5.0.

### Immunoprecipitation and Immunoblotting (IPIB)

A549 cells were cultured to 90% confluency prior to serum starvation for 24 hours. Protein phosphatase inhibitors, sodium orthovanadate (2mM), and sodium fluoride (1 mM) were added for 2 hours prior to the addition of rspike RBD (100ng/plate). After 12 hours of incubation, cold RIPA buffer was added, and the cells were lysed. For immunoprecipitation, an equal concentration of protein (1mg) from test and control lysates was treated with an anti-Flt-1 antibody (10µg, overnight at 4°C) prior to adding protein A agarose beads. The complex was centrifuged at 4000 rpm for 5 minutes and washed prior to processing for SDS-PAGE and western blotting. The blot was probed with either anti-spike RBD antibody or anti-Flt-1 antibody. Further, the blot was washed and incubated with goat anti-rabbit HRP tagged secondary antibody followed by ECL development. rspike RBD-induced phosphorylation of proteins in A549 cells was assessed using rspike RBD treated A549 cell lysates by SDS-PAGE and western blotting. The blot was probed with anti-p-Tyr- antibody to visualize the phosphorylated proteins.

### Immunofluorescence Assay Using MDA-MB 231 Cells in Either 2D- or 3D- Spheroid Culture

MDA-MB 231 cells were grown in L-15 media on sterile glass coverslips for 24 hours and were treated either with rspike RBD, rsFlt-1 alone, or a pre-incubated mixture of rspike RBD and rsFlt-1 for 2 hours at 37°C. MDA-MB 231 cell-spheroids were generated using 2000 cells/spheroid in L-15 media containing 0.25% methylcellulose. Spheroids were treated with rspike RBD, either alone or with rsFlt-1, rVEGF, either alone or with rsFlt-1 for 2 hours at 37°C. Either cell in 2D- or spheroid cultures were fixed using 4% paraformaldehyde and blocked with 3% bovine serum albumin (BSA). The cells or spheroids were probed with primary antibodies (rabbit polyclonal anti-sFlt-1, anti-spike RBD, or anti-VEGF at 1:100 dilution overnight at 4°C) prior to the addition of goat anti-rabbit IgG Alexa Flour 488- tagged secondary antibody (1:500 dilution) and incubation in the dark at room temperature for 1 hour. Cells in 2D culture were labeled with Alexa Flour 594 Phalloidin. Cells or spheroids were stained with DAPI or Hoechst respectively prior to mounting with coverslips using VECTASHIELD and imaging. Image acquisition and analysis were made using a confocal microscope (Carl Zeiss, Germany) with ZEN blue 2010 software.

### Statistical Analysis

All results are represented as the mean ± SD for all values obtained in triplicate from three independent tests. Statistical tests for normal distribution were conducted by D’Agostino & Pearson test, the Anderson-Darling test, or the Shapiro-Wilk test and conform to normal distribution. Further two-way ANOVA (parametric) using Tukey or Dunnett’s multiple comparisons test was chosen for multiple comparisons between multiple groups including test or hypothesis and control groups. One-sample t-test (two-tailed) was used to compare between test and control groups in the case of two groups comparison (**Figure 4C**). GraphPad Prism 8 software was used to perform all statistical analyses. **** P<0.0001, *** P < 0.001; ** P < 0.01; and * P < 0.05 was used to indicate statistically significant groups.

## Results

### Structural Similarity Between Spike-RBD and sFlt-1 Proteins

**Figure 1:**
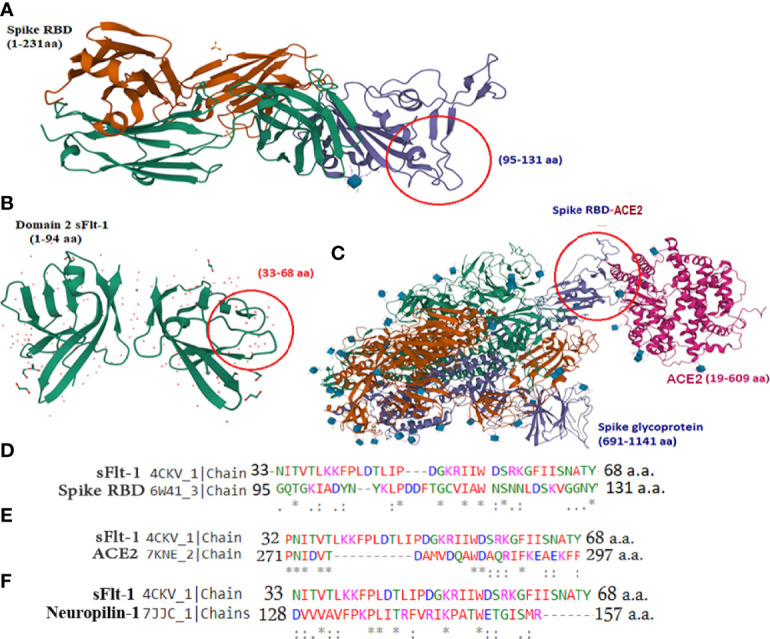
3D structure similarities between sFlt-1 and SARS-CoV-2 spike-RBD: **(A)** SARS-CoV-2 spike RBD structure (PDB, 6W41). The RBD domain is highlighted in purple color and amino acid residues 95–131 is highlighted in red circle. **(B)** 3D structure of domain-2 sFlt-1 (4CKV), red circle highlights the amino acid residues 33-68 with similar properties of the spike-RBD amino acid residues (95-131). **(C)** 3D structure of SARS-CoV-2 spike RBD (purple colour) bound with ACE2 (7KNE), pink colour **(D)** Sequence alignment of 33-68 amino acid residues of sFlt-1 with 95-131 amino acid residues of spike RBD. **(E)** Sequence alignment of 32-68 amino acid residues of sFlt-1 with 271-297 amino acid residues of ACE2 (7KNE). **(F)** Sequence alignment of 33-68 amino acid residues of sFlt-1 with 128-157 amino acid residues of neuropilin-1 (7JJC_1). All structures were retrieved from PDB InterPro.

**Figure 2:**
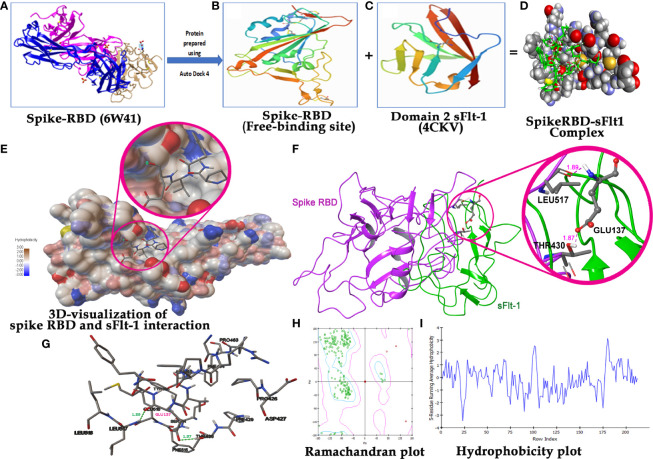
Molecular Docking analysis demonstration binding of sFlt-1 to spike RBD: **(A)** 3D structure of spike RBD retrieved from PDB (6W41) and prepared for docking. **(B)** Spike RBD 3D-structure (333-525 amino acid residues) after deleting extra chains. **(C)** 3D structure of domain 2 sFlt-1 retrieved from PDB (4CKV) and prepared for docking (132-225 amino acid residues) as a ligand. **(D)** Spike RBD -sFlt-1 complex as a result of autodock run analysis. **(E)** 3D visualization of spike RBD and sFlt-1 interaction with close view showing the binding of sFlt-1 amino acid residues in the active site groove of spike RBD. hydrophobicity scale represents colours (hydrophilic to hydrophobic) shown in the active site of spike RBD. **(F)** Ribbon representation of spike RBD and sFlt-1 interaction showing the binding of Glu137 of sFlt-1 with Leu517 and Thr430 of spike RBD by two hydrogen bonds. **(G)** 2D-visualization of the protein-protein interaction showing the amino acids involved in this binding. **(H)** Ramachandran plot demonstrating the plot of the torsional angles - phi (φ) and psi (ψ) - of the amino acid residues contained in a protein. plotting the φ values on the x-axis and the ψ values on the y-axis shows which combination of angles is possible. The torsional angles of each residue in a peptide determine the conformation of the residues and the protein. **(I)** Hydrophobicity plot: Indicates the amino acids in a complex protein against their hydrophobicity index, residues with hydrophobicity of 0.7 or more are considered hydrophobic and those under -2.4 are hydrophilic.

It has been reported in the literature that increased sFlt-1/PlGF ratio in COVID-19 is a novel link to angiotensin II-mediated endothelial dysfunction (21). In addition, many studies (16, 22, 23) have reported that Covid positive patients with high sFlt-1 levels have extensive endothelial damage characterized by thrombotic complications, and a poor prognosis. Since sFlt-1 contains regions with sequence identities with spike-RBD, as reported in **Figure 1D**, we compared the 3D structures of these two proteins to verify whether their structural similarity could provoke the cross-reactivity of antibodies specific for each protein for the other one as shown in **Figure 1**. We found that the two proteins show similarity in the regions including anti-parallel β-sheets surrounded by two α-helices, evidenced by the red circles in the **Figures 1A, B**, containing common sequences corresponding to 95–131 amino acid (a.a) residues in spike RBD (**Figure 1A**) and 33–68 aa residues in the domain two of sFlt-1 protein (**Figure 1B**). In **Figure 1C**, the N-terminal helix of ACE2 possess 20 amino acid residues that interact with 16 amino acid residues of SARS-CoV-2 RBD, among them 17 amino acid residues are shared (24). Other homologies between sFlt-1 and spike RBD, have been reported in the spike RBD and sFlt-1 domains sequence alignment (**Figure 1D**). Data in **Table 1** (**Supplementary Data**) indicate the similarity in the position of specific amino acid residues in 2domain sFlt-1 (4CKV_1) sequence 33-68 and spike RBD (6W41) 95- 131 amino acid residues respectively. Additionally, sequence alignment of sFlt-1 and known spike RBD binding proteins e.g., ACE2 and neuropilin-1 revealed sequence homology as shown in **Figures 1E, F** respectively. So, similarity in the 3D-structure of the two proteins in these particular regions leads to antibody cross reactivity between the two proteins and these findings has been confirmed by ELISA studies in **Figure 4A** as reported that spike RBD can be recognized by anti-Flt-1 antibody. Further to the similarities in the structures, our hypothesis on the interaction of spike RBD with Flt-1 receptor was also validated using molecular docking studies. The domain two of sFlt-1 protein was investigated as a ligand for the SARS-CoV-2 spike RBD protein. Docking and simulation studies reported a ligand-receptor binding of the two proteins (**Figures 2A–D**). In this study, docking simulation has been performed using Auto Dock Tools 4.2 program. The detailed results of docked score are represented in **Table 1**. sFlt-1 exhibited significant hydrogen bond interactions at the active binding site of spike RBD, i.e., with oxygen group atom (GLU137) of sFlt-1 and amino group atom of LEU517 of spike RBD, and an oxygen atom (GLU137) of sFlt-1 with the hydrogen atom of THR430 of spike RBD recording the lowest binding energy of -5.03 kcal/mol at a distance of 1.89 Å and 1.87 Å respectively, as shown in **Figures 2F, G**. Also, other putative amino acids of sFlt-1 are Pro134:O, Arg133:O, Gly132:O, Met138:O, and Gly139:OH involved in binding spike RBD with Asp427:HN, Phe377:HN, Thr430:HN, Thr430:HG1, and Leu517:HN respectively. 3D visualization of spike RBD and sFlt-1 interactions with hydrophobicity binding scale was reported as well as shown in **Figure 2E**. **Figure 2H** confirmed the torsional angles involved in the interaction of each amino acid residue of the ligand (sFlt-1) and the protein (spike RBD), Further, it has been shown that the amino acids involved in the interaction between the two proteins are having hydrophobic properties (**Figure 2I**).

**Table 1:** Summary of molecular docking score results of spike RBD with domain 2 of sFlt-1 obtained by Auto dock 4.

Binding Energy (kCal/mol)	Ligand efficiency	Inhibition constant, µM T = 298.15 K	vdW- hb- desolv-energy kCal/mol	NO. of Hydrogen bonds with amino acids	Bond length (Å)	RMSD(Å)	
						Cluster	Reference
**-5.03**	-0.07	205.52	-13.75	THR430:HG1:GLU137:OE1 LEU517:HN : GLU137:O	1.89 1.87	0.0	29.25

### Confirmation of rspike (RBD) and rsFlt-1

**Figure 3:**
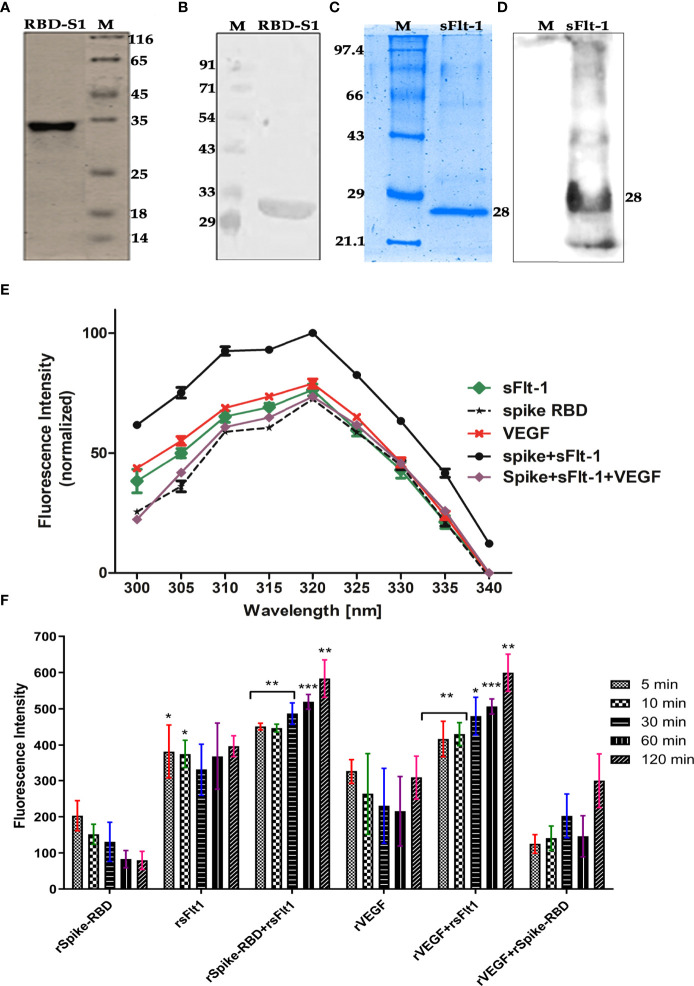
**(A–D)** Confirmation of purified rspike RBD and rsFlt1 proteins by SDS-PAGE and western blotting. **(A)** Silver-stained gel image of purified His−tagged recombinant SARS−CoV−2 spike (RBD-S1) protein; Lane (1) rspike RBD protein, Lane (2) Unstained Protein Molecular Weight Marker. **(B)** Western blot image of purified rspike RBD-S1 Protein; Lane (1) Pre-stained protein molecular weight marker, Lane (2) rspike RBD protein with molecular weight of ~33 Kda. **(C)** Coomassie brilliant blue stained gel of purified rsFlt-1; Lane (1) Unstained protein molecular weight marker. Lane (2) rsFlt-1. **(D)** rsFlt-1 purification confirmed on western blot; Lane (1) pre-stained protein molecular weight marker, Lane (2) rsFlt-1 protein with molecular weight of 28Kda. M denotes for molecular weight marker **(E)** Fluorescence scan of intrinsic amino acids revealed rspike RBD+rsFlt-1 interaction with highest fluorescence intensity, VEGF, anti-Flt-1 antibody and anti-spike RBD antibody used as competitors or inhibitors of the interaction respectively, Error bars too small and data represented as Mean ± SEM. (n=3). **(F)** The fluorescence intensity of rSpike RBD/sFlt-1 alone or in combination showing protein-protein interactions at time-dependent manner based on the fluorescent aromatic amino acids, rVEGF-rsFlt-1 or rVEGF-rspike RBD are positive and negative controls respectively. The fluorescence emission by rspike RBD-rsFlt-1 or rVEGF-rsFlt-1 complexes was comparatively higher than the fluorescence emitted by rSpike RBD, rsFlt-1, or rVEGF alone. Statistical test for normal distribution was conducted by D’Agostino & Pearson test and Anderson-Darling test conforms normal distribution. Further two-way ANOVA (parametric) using Dunnett’s multiple comparisons test was chosen for multiple comparisons between spike RBD+sFlt-1 and spike RBD +sFlt-1 + VEGF revealed a significant difference (P<0.0001). In Panel F, Shapiro-Wilk test was applied for the normal distribution test and confirms the normality of the data, two-way ANOVA by Dunnett’s multiple comparisons test, was further used for the comparison of multiple groups. P-values indicates statistical significance compared to control groups (*P<0.05, ** P<0.01, *** P<0.001).

It was revealed that rspike RBD and rsFlt1** **were successfully expressed in bacterial strains, Rosetta (DE3) and *Escherichia coli* (BL21 strain), respectively. Expressed proteins purified by metal ion affinity chromatography (Ni-NTA) showed more than 98% purity as per SDS-PAGE and western blot analysis. Validation of rspike (RBD), with a molecular weight of 35kDa, was confirmed using anti-spike antibodies (**Figure 3A**) from serum samples obtained from SARS-CoV-2 (Positive) individuals. Likewise, antibody to sFlt-1 confirmed rsFlt-1 with a molecular weight of 28kDa (**Figures 3C, D**). Purified expressed recombinant sFlt-1 and spike RBD proteins obtained from three expression batches were quantified and expressed as a representative plot with Mean ± SEM, (n=3) as shown in supplementary data **Figures 3A, B**.

The Protein-protein interaction between rspike RBD and rsFlt-1 is determined by using fluorescence emission by respective proteins alone or in combination. The results revealed that** **upon the interaction of the two proteins, there was an increase in the emission compared to either of the proteins alone. The data in **Figure 3E** reveals characteristic emission spectra of rspike RBD or rsFlt-1 or the two interacted proteins rspike RBD and rsFlt-1 complex. VEGF was used as competitor or inhibitor of the spike RBD and sFlt-1 interaction revealed a significant decrease in the fluorescence intensity as compared with spike RBD and sFlt-1 complex, data represented as Mean ± SEM. (n=3). Furthermore, the fluorescence intensity of rspike RBD/sFlt-1 alone or in combination revealed protein-protein interactions in a time-dependent manner, based on the fluorescent aromatic amino acids. We used rVEGF-rsFlt-1 or rVEGF-rspike RBD as positive and negative controls, respectively. The fluorescence emission by rspike RBD-rsFlt-1 or rVEGF-rsFlt-1 complexes was comparatively higher than that emitted by rspike RBD, rsFlt-1, or rVEGF alone (**Figure 3F**).

### Analysis of the Interactions of rspike RBD, sFlt-1, and the Cross-Reactivity of Their Antibodies for the Two Proteins

**Figure 4:**
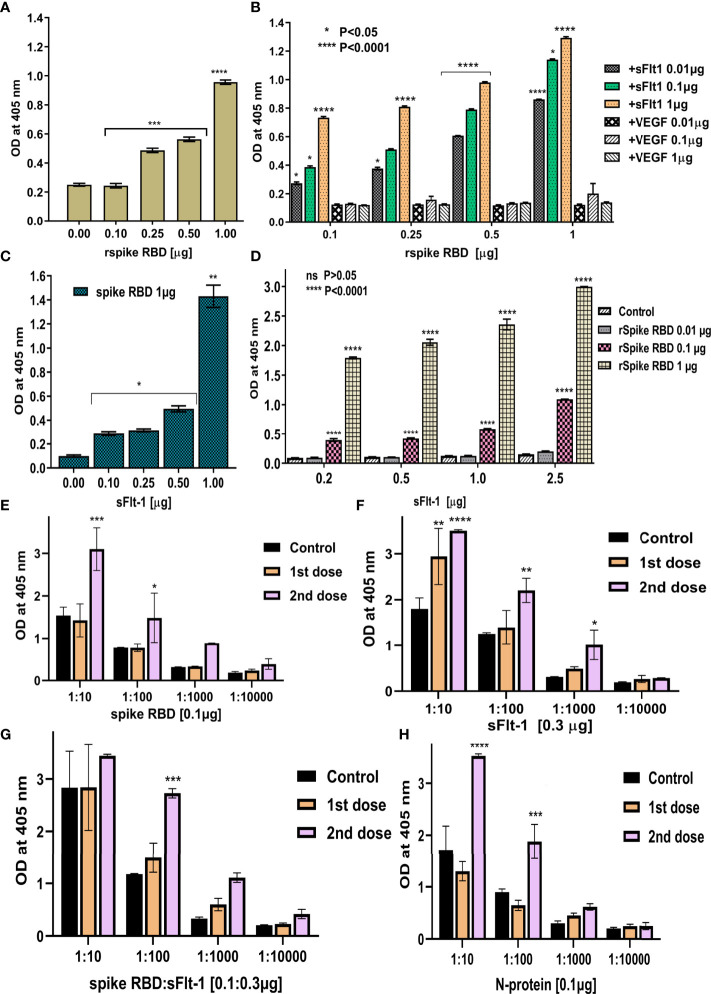
Demonstrating the binding between rspike RBD and rsFlt-1 using ELISA: **(A)** Binding of anti-sFlt-1 antibody to spike-RBD immobilized on the ELISA plate, control is without spike RBD immobilization. It confirms normal distribution by both Shapiro-Wilk and Kolmogorov-Smirnov test and significant deviation from zero (P<0.0001) was observed by simple linear regression test. **(B)** Binding of serial dilutions (0.01,0.1, and 1µg) of rsFlt-1 to increased concentrations of rspike RBD immobilized on the ELISA plate and probed with polyclonal sFlt-1 antibody. Addition of VEGF to the wells immobilized with spike RBD instead of sFlt-1 was used as control. **(C)** Binding of serial dilutions of rsFlt-1 (0.1, 0.25, 0.5 and 1µg) immobilized on ELISA plate to spike RBD (0.1 µg) which further probed with anti-spike antibody. Control is without sFlt-1 immobilization. One-sample t test (two-tailed) was used to compare between test and control group revealed significant increase in signal upon spike RBD addition (P value =0.0036). **(D)** Binding of serial dilutions of rsFlt-1 (0.2, 0.5, 1 and 2.5 µg) immobilized on ELISA plate to increased concentrations of spike RBD (0.01, 0.1 and 1µg) which further probed with anti-spike antibody. Wells coated with VEGF were used as control. **(E–H)** ELISA assays to test the cross-reactivity for rspike RBD, sFlt-1, spike RBD-sFlt-1, or nucleocapsid protein immobilized on the ELISA plates to the human anti-spike polyclonal antibodies (1:10, 1:100, 1:1000 and 1:10000 dilutions) from vaccinated donors (dose 1 and 2) versus non-vaccinated donors as a negative control. Error bars depicted mean SEM ± SD, Statistical test for normal distribution of panels **(B, D)** using Shapiro-Wilk test confirms normality. Further, two-way ANOVA was used for multiple groups comparison by Tukey and Dunnett’s multiple comparisons tests in panels **(B, D)** respectively. Panels **(E–H)** confirms normal distribution by Shapiro-Wilk test followed by two-way ANOVA Dunnett’s multiple comparisons test was applied to compare between control (non-vaccinated) versus1^st^ and 2^nd^ dose of vaccinated serum, P-values indicate statistical significance compared to control groups (*P<0.05, ** P<0.01, *** P<0.001, **** P<0.0001).

Further to the above findings, the ELISA data in **Figure 4A** shows that the immobilized spike RBD protein is recognized by anti-sFlt-1 antibody. When different concentrations of spike RBD at 0.1, 0.25, 0.5, and 1.0 µg were immobilized, the polyclonal anti-sFlt-1 antibody recognizes the immobilized spike RBD even at a lower concentration (0.1 µg), as shown in **Figure 4A**. To measure the binding affinity of rspike RBD to rsFlt-1, results of sandwich ELISA revealed successful capture between the immobilized rspike RBD and rsFlt-1 proteins at dose-dependent response recognized by the polyclonal anti-sFlt-1 antibody, a significant signal reaching saturation at the low concentration of 0.01µg was observed, the data was also compared with control where different concentrations of VEGF (a protein that doesn’t bind to spike RBD) was used, there was no recognition of VEGF- spike RBD complex either by anti-sFlt-1 antibody or anti-VEGF antibody as shown in **Figure 4B**. Furthermore, when different concentrations of sFlt-1 were immobilized and rspike RBD was added (1µg) and further probing using polyclonal anti-spike RBD revealed a dose-dependent increase in the absorbance with increasing concentration of immobilized sFlt-1 as against the control value where sFlt-1 was not immobilized on the plate (**Figure 4C**). In **Figure 4D** different concentrations of sFlt-1 (0.2, 0.5, 1.0, and 2.5 µg) were immobilized and captured with increasing concentrations of rspike RBD (0.01, 0.1, and 1.0µg) for verification of binding interaction between the two proteins using spike RBD monoclonal antibody to detect these interactions. The data reveals that significant binding was detected and the response was in a dose-dependent manner, this result was compared with an appropriate control where VEGF was immobilized and spike RBD used as a binding partner then developing with anti-spike RBD antibody does not reveal any interaction as shown in **figure 4D**. To further confirm these data, we also tested the cross-reactivity of sFlt-1 proteins to plasma samples obtained from vaccinated donors, yoga practitioners containing high levels of human anti-spike antibodies by ELISA assays. The anti-spike RBD antibodies produced by the vaccine injection show significant binding to either spike RBD or sFlt-1 immobilized on the ELISA plate (vaccinated donors), as the signal intensity was comparable to that observed for the negative control (non-vaccinated donors) as shown in **Figures 4E, F**. The spike RBD and sFlt-1 binding was further recognized by human anti-spike antibodies from vaccinated donors which results in a significant increase in the absorbance as shown in **Figure 4G**. These findings suggest that the anti-spike RBD antibodies induced by vaccination do recognize sFlt-1 or spike RBD-sFlt-1 complex. Thus, they are binding partners, and the Flt-1 receptor could be another binding receptor of spike RBD on the host cell.

**Figure 5:**
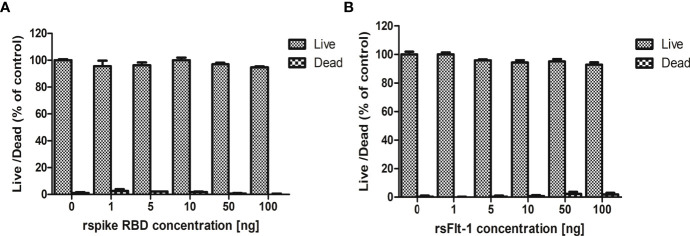
Assessment of cytotoxicity of rspike RBD and rsFlt-1: **(A)** A549 cells treated with rsFlt-1 or **(B)** rspike RBD at 1, 5, 10, 50, 100 ng revealed no significant cytotoxic effect on treated cells compared to control. The experiment was performed in triplicate; SD and error bars are depicted in the graph.

The effect of rspike RBD or rsFlt1 on the viability and proliferation of lung cell line A549 indicated that these two proteins did not compromise and were not cytotoxic at different concentrations (1,5,10, 50,100 ng) used for the study (**Figure 5**).

**Figure 6:**
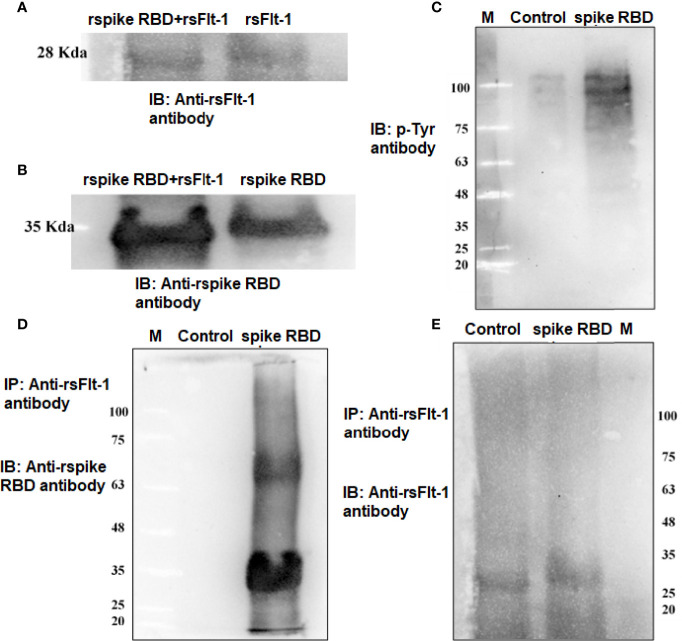
Detection of binding of rspike RBD with cell surface Flt-1 receptors using immunoprecipitation and immunoblotting: **(A)** Lane1 is immunoblot of rspike RBD-rsFlt-1 complex (pre-incubated for 2-hours prior to run on the gel) and lane 2 is rsFlt-1 protein, both lanes probed with anti-sFlt-1 antibody. **(B)** Immunoblot of rspike RBD- rsFlt-1 complex (lane 1) and rspike RBD (lane 2) probed with anti-Spike RBD antibody. **(C)** Immunoblot of untreated and rspike RBD treated cell lysate probed with p-Tyr antibody indicates more phosphorylation in treated lysate compared to control. **(D)** IP of rspike RBD of A549 treated cell lysates with anti-sFlt-1 antibody followed by IB with anti-spike RBD antibody shows clear bands in treated lane compared to the absence of bands in untreated cells revealed precipitation of cellular bounded spike RBD by the anti-sFlt-1 antibody. **(E)** IP of cell lysates using sFlt-1 antibody and IB with anti-sFlt-1 antibody showing bands in both treated and untreated cells due to presence of sFlt-1 receptor on the cells regardless of the treatment.

**Figure 7:**
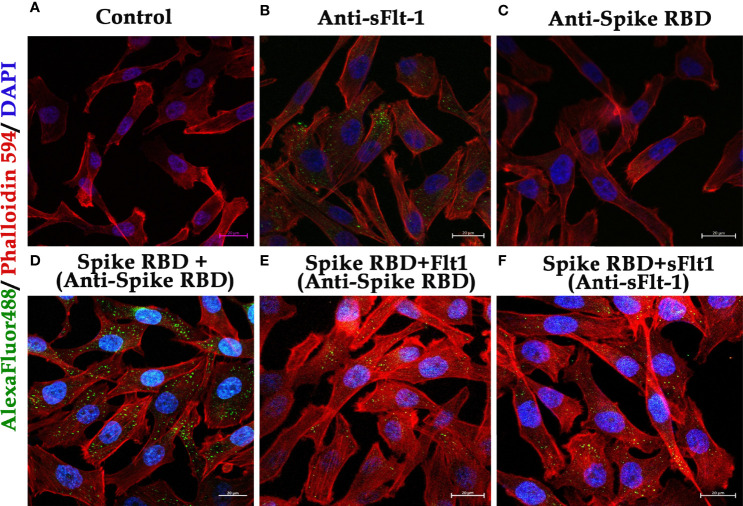
Immunofluorescence of MDA-MB 231 cells in 2D culture to demonstrate binding of rspike RBD to Flt1-1 receptors: **(A)** Control (cells only without any treatment) **(B)** Cells probed with anti-Flt-1 antibody. **(C)** Cells probed with anti-spike RBD antibody. **(D)** Cells were treated with rspike RBD and probed with anti-spike RBD. **(E, F)** Cells were treated with a pre-incubated rspike RBD and rsFlt1 and probed with either anti-spike RBD or anti-sFlt-1 respectively, which shows a lack of fluorescence signals as a result of binding of the two proteins during pre-incubation. The scale bar is present on each captured image, indicating 40X magnification.

**Figure 8:**
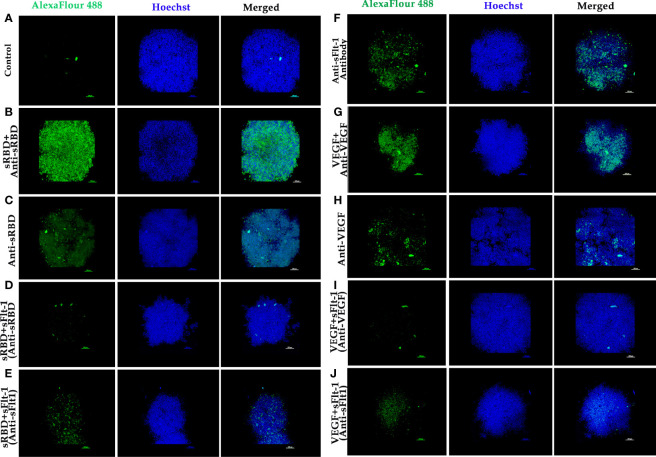
Immunofluorescence of 3D spheroids of MDA-MB 231 cells to demonstrate binding of rspike RBD or rVEGF to Flt-1 receptors: **(A)** 3D spheroid (control). **(B)** Spheroids were treated with rspike RBD and probed with anti-Spike RBD antibody followed by goat anti-rabbit AlexaFlour 488 secondary antibody showing strong fluorescence signal (green fluorescence) which indicates binding of spike RBD to cellular receptors, Hoechst was used for nuclei staining (blue fluorescence). **(C)** Spheroids probed with anti-Spike RBD antibody. **(D, E)** Spheroid was treated with pre-incubated rspike RBD and rsFlt-1 mixture and probed either with anti-Spike RBD or anti-Flt1 antibody (no fluorescence signals were reported revealing spike RBD and sFlt-1 interaction in the incubation period prior to adding the complex to the spheroids. **(F)** Spheroid probed with anti-sFlt-1 antibody shows fluorescence signals due to binding of sFlt-1 antibody to Flt-1 receptors on the cells. **(G)** Spheroid was treated with rVEGF and probed with an anti-VEGF antibody shows fluorescence signals due to binding of VEGF antibody to VEGF on the cells. **(H)** Spheroid probed with anti-VEGF antibody. **(I, J)** Spheroid was treated with pre-incubated rVEGF and rsFlt-1 mixture and probed either with anti-VEGF or anti-Flt-1 antibody leads to loss of fluorescence signals which may be as a result of interaction between VEGF and sFlt-1 during incubation period prior to adding the mixture into the spheroids. The scale bar indicates 10X magnification.

The data on western blot analysis in **Figures 6A, B** indicates the cross-reactivity between recombinant proteins (sFlt-1 or spike RBD) with their respective antibodies. After the validation for the interaction of specific antigens (sFlt-1 or spike RBD) with their respective antibodies, we have used these antibodies for our further experiments of immunoprecipitation and immunoblotting (IPIB). Because our data on *in silico* and spectrofluorimetric analysis indicated a clear interaction between pure rspike RBD and rsFlt-1, we have further undertaken binding studies at the cellular level. The results shown in **Figure 6** indicate that the addition of rspike RBD to A549 cells successfully binds to cellular Flt-1 receptors. The specific binding between spike RBD and cellular Flt-1 receptor could be captured by immunoprecipitating the complex with an anti-Flt-1 antibody. The tri-parted complex (Spike RBD, Flt1 receptors plus anti-Flt1 antibody) was recognized by anti-spike antibody as visualized in data on western blotting shown in **Figure 6**. Immunoblot of untreated (control) and rspike RBD treated cell lysate probed with p-Tyrosine antibody revealed phosphorylation steps during binding of rspike RBD to its receptors on the cell membrane, as shown in **Figure 6C**. Immunofluorescence data in control cells (**Figure 7A**), shows no immunofluorescence detected. While, as shown in **Figure 7B**, data of immunofluorescence studies revealed that MDA-MB231 cells highly express Flt-1 receptors and could be detected using anti-Flt-1 antibody by AlexaFlour488 tagged secondary antibody (green fluorescence). However, in **Figure 7C** no immunofluorescence was detected when cells were probed with an anti-spike RBD antibody. Subsequently, **Figure 7D** indicates that the rspike RBD binds to its cellular receptors (ACE2 or Flt-1), which can be detected using an anti-spike antibody. In **Figures 7E, F**, it is evident that there is no detectable immunofluorescence signal on MDA-MB231 cells when rspike RBD and Flt-1 have interacted by pre-incubation prior to adding the same to the cells, which confirmed capturing of rspike RBD by rsFlt-1. In **Figure 8**, immunofluorescence data on 3D spheroid culture of MDA-MB231 cells reveals no immunofluorescence signals were detected in either control spheroids (**Figure 8A**) or spheroids probed with anti-spike RBD antibodies (**Figure 8C**). While the intensity of immunofluorescence in spheroids treated with rspike RBD (**Figure 8B**) and then probed with anti-spike RBD antibody was significantly evident. In **Figures 8D, E**, the result clearly indicates that when rspike RBD was captured with rsFlt-1 prior to treating the spheroids there was no significant immunofluorescence detected as compared to **Figure 8B**. As 3D spheroids of MDA-MB 231 cells express VEGF receptors, spheroid probed with anti-sFlt-1 antibody (**Figure 8F**), VEGF (**Figure 8G**), or anti-VEGF antibodies (**Figure 8H**) used as a positive control and revealed high-intensity immunofluorescence signals on 3D-spheroids. Spheroids treated with interacted rVEGF and rsFlt-1 proteins by pre-incubation and probed with either anti-VEGF or anti-Flt-1 antibody lack fluorescence signals and confirm the capture of VEGF receptors by sFlt-1 protein (negative control -**Figures 8I, J**).

## Discussion and Conclusion

Here, we are reporting results of *in silico* screening and *in vitro* assays intended to identify the anti-inflammatory and binding affinity of the sFlt-1 molecule, which may have implications in the prevention and/or treatment of the SARS-CoV-2 infection. With a prerequisite to identify effective therapies to test, prevent or treat COVID19 infection rapidly, we initiated *in silico* screening to identify potential molecules that can be used to inhibit or divert the interaction of the Spike (RBD) protein, with the help of which the SARS-CoV-2 virus is reported to hijack the cellular machinery of host cells (25). Spike RBD has been identified as the most probable pharmacological target to date. These spike proteins also elucidate an antibody response. Therefore, understanding antigen-binding epitopes may aid in developing vaccines and medications against the COVID19 pandemic (26). Structural similarities of the spike RBD and sFlt-1 have led to the binding of the proteins or their antibodies to each other. Similar structural similarities and interaction findings between Spike of SARS-CoV-2 and platelets factor 4 (PF4) were reported in the literature (27). Molecular docking has proven to be a useful approach for discovering and optimizing novel lead molecules against specific targets (28). The binding affinity between the ligand and its target receptor is reflected in the docking score. The higher the negativity in the binding energy, the more potent the medication is (29). In addition to computational findings (*in silico*), fluorescence spectroscopy gives information about the interactions occurring within the protein’s peptide chains and is also a well-known method for characterizing the structural conformation of proteins (30, 31),. The method is based on the intrinsic fluorescence properties of three aromatic amino acid residues of protein: phenylalanine, tyrosine, and tryptophan. Emission spectra of most of the proteins are dominated by tryptophan (Trp), which emits at the longest wavelength and has the highest extinction coefficient and quantum yield. Moreover, the excitation energy of tyrosine (Tyr) residues can also be transferred to Trp residues. The sensitivity of Trp fluorescence to its immediate surroundings is an intriguing trait. As a result, variations in Trp emission spectra can be linked to changes in tertiary structure of a protein, such as conformational shifts, subunit interaction, substrate binding, or denaturation (31). Our data on the spectrofluorimetric analysis of bound versus individual protein (spike RBD vs. sFlt-1), showed an increase in emitted fluorescence upon the interaction of the two proteins. This may be a consequence of confirmational changes in these three aromatic amino acids (tryptophan, phenylalanine and tyrosine) can be captured by spectrofluorometer, indicating high emission fluorescence due to conformational changes (20). Furthermore, immunoprecipitation assay with whole cell lysate confirms these interactions where proteins present in their native form in a complex mixture of cellular components were precipitated with anti-Flt-1 antibodies and separated by agarose beads for SDS-PAGE and immunoblotting with anti-spike RBD antibody that confirms successful interactions. At the cellular level, SARS-CoV-2 exploits human ACE2 for host cell entry. CD147/S-protein-mediated route of invasion has also been reported (32). Viral entry depends on the binding of the S1 subunit to ACE2 through the receptor-binding domain RBD in the S1 subunit, facilitating viral attachment to the surface of target cells. ACE2 represents a significant target for inhibiting COVID19 infection by disturbing the regulation of immune competence, inflammatory equilibrium, tight junctional barriers, hemodynamic stability, as well as thrombotic and fibrinolytic pathways (33). ACE2 receptors are considered for the management of COVID19 outbreaks (34). COVID 19 infection is considered a vascular disease where there is dysregulation and dysfunction of the vascular system (35). The severity of COVID 19 infection leads to the derailment of vascular homeostasis (36). The early onset of viral replication induces vascular leakage and massive apoptosis of epithelial and endothelial cells, triggering the release of pro-inflammatory cytokines and chemokines (37), including VEGF. It has been reported in the literature that an increased sFlt-1/PlGF ratio in COVID-19 is a novel link to angiotensin II-mediated endothelial dysfunction (21). In the current study, we have used molecular docking to discover and optimize the binding of spike RBD with specific target proteins having a role in vasculo- and angiogenesis. Recently, some compounds that directly bind to the ACE2 receptor with high affinity are suggested for competing with the virus, e.g., morphine and codeine (38). Studies have revealed cytokine storms also contribute to brain inflammation and trigger neurological manifestation during COVID 19 infection. VEGF is widely distributed in the brain and is known to play a crucial role in brain inflammation by facilitating the recruitment of inflammatory cells and regulating the levels of angiopoietin (39, 40). VEGF-targeted drugs may have therapeutic potential in suppressing inflammation during COVID19 infection with neurological symptoms (41). VEGFR2, also known as Flk1, binds to VEGF A and controls angiogenesis in healthy and diseased tissues. VEGFR1, also known as Flt-1, binds to VEGFA, VEGFB, and PlGF. The tyrosine kinase activity of Flt-1 contributes to inflammation (42). The data presented in this work shows that recombinant 2-domain soluble Flt-1 (2d-sFlt-1) binds to rspike RBD. We have validated specific interactions between the two proteins using *in silico* computational studies, spectrofluorimetric, and immunoassays such as ELISA and immunofluorescence studies. Our data have indicated that the aromatic amino acids in the second domain of sFlt-1, which bind to the active site of spike RBD, have homology with the amino acids of ACE2 that are involved in the interaction of ACE2 with spike RBD. Our data on experiments using ELISA indicate that there is effective and specific recognition, binding, and interaction between rspike RBD and rsFlt-1, over a range of concentrations used in ELISA. A good antibody titter in vaccinated serum from healthy volunteers further strengthened our ELISA data. We noticed that the anti-Flt-1 antibody recognizes rspike RBD, confirming the similarity of spike RBD and sFlt-1 3D structures obtained in our bioinformatics studies (**Figure 1**) therefore indicating that rspike RBD and rsFlt1 may have similarity in epitopes recognized by the anti-Flt-1 antibody. To the best of our knowledge, this is the first report showing the binding of rspike RBD with Flt-1 cell surface receptor. Our data on both immunofluorescence and IPIB reveal specific binding of spike RBD to cell surface Flt-1, where an increase in fluorescence and positive cross-reactivity with respective antibodies in immunoblotting experiments further confirmed clear interaction between spike RBD and cell surface Flt-1 receptors. Such interactions may trigger intracellular phosphorylation of specific proteins, especially on tyrosine residues, as is evident from our data. Our data of immunofluorescence studies indicate that the rspike RBD binds to its cellular receptors (ACE2 or Flt-1), which can be detected using an anti-spike antibody. It was further observed that there wasn’t any detectable immunofluorescence signal when rspike RBD and rsFlt-1 were pre-incubated prior to adding the same to the cells, which confirmed capturing of rspike RBD by rsFlt-1. Likewise, in 3D spheroid culture, there wasn’t any immunofluorescence on the spheroids exposed to a pre-incubated mix of Flt-1 and spike RBD, while there was immunofluorescence when spheroids were exposed to spike RBD alone, indicating an interaction of rspike RBD with cell surface receptors such as Flt-1 receptors. Spheroids treated with a pre-incubated/interacted mix of rVEGF and rsFlt-1 proteins and probed with either anti-VEGF or anti-Flt-1 antibodies lack fluorescence signals, thus confirm the capture of VEGF receptors by sFlt-1 protein (negative control). The molecular mechanism proposed in this study could represent the pathogenetic basis of the previously reported clinical observations (16, 22, 23) that hospitalized Covid positive patients diagnosed with high sFlt-1 levels and have extensive endothelial damage characterized by thrombotic complications, and a poor prognosis. From all the above findings, we conclude that spike RBD interacts with the cellular Flt-1 receptor to elicit signal transduction and regulation of specific cellular pathways mediating the pathogenesis of COVID19 infection. Further, such binding of spike RBD with Flt-1 receptor will interfere with VEGF-Flt-1 access towards cellular signaling and angiogenesis, as is evident in COVID19 infection.

## Data Availability Statement

The original contributions presented in the study are included in the article/**Supplementary Material**. Further inquiries can be directed to the corresponding author.

## Ethics Statement

The animal study was reviewed and approved by the Institutional Animal Ethics Committee/ CPCSEA/ IAEC, New Delhi with an approval number UOM/IAEC/23b/2018. 

## Author Contributions

Senior Authors: BS (Principal Investigator) and PS; Conception and design of the research work, data analysis and interpretation of the results, drafting of the manuscript, critical editing and revision of the manuscript. First Author: AM; Conducting and reporting of experiments, critical data collection, production of recombinant Flt-1 and anti-Flt-1 antibodies, drafting and revision of the manuscript. Co-authors: MD, DS, and RD; Production of recombinant spike RBD protein and anti-spike RBD antibodies, recombinant VEGF and anti-VEGF antibodies and ELISA experiments, revision of manuscript. All authors contributed to the article and approved the submitted version.

## Funding

BS acknowledges the financial support from the department of science and technology government of India (SR/SATYAM/424/2017-2018).

## Conflict of Interest 

Author RD is employed by PS (Director of Sanorva Biotech. Pvt. Ltd.), MD, and DS are directors of Denovo Biolabs Pvt. Ltd.

The remaining authors declare that the research was conducted in the absence of any commercial or financial relationships that could be construed as a potential conflict of interest.

## Publisher’s Note

All claims expressed in this article are solely those of the authors and do not necessarily represent those of their affiliated organizations, or those of the publisher, the editors and the reviewers. Any product that may be evaluated in this article, or claim that may be made by its manufacturer, is not guaranteed or endorsed by the publisher.
