# Supplementary material for: Vascular Endothelial Growth Factor Receptor, fms-Like Tyrosine Kinase-1 (Flt-1), as a Novel Binding Partner for SARS-CoV-2 Spike Receptor-Binding Domain
Source: Front Immunol. 2022 Jul 8;13:906063. doi: 10.3389/fimmu.2022.906063 (PMC9304886; doi:10.3389/fimmu.2022.906063)
Supplement: Supplementary file 1 [file DataSheet_1.pdf]

RESULTS

In Figure 1, We have compared the 3D structures of the two proteins (sFlt-1 and spike RBD) to verify whether their structural similarity could provoke the cross-reactivity of antibodies specific for each protein for the other one. Sequence homology between 2domain sFlt-1 (4CKV_1) 33-68 and spike RBD (6W41) 95- 131 amino acid residues reveals amino acid residues with similar properties present in both the proteins in specific positions as shown in figure 1D and table 1. We found that the two proteins show similarity in the regions including anti-parallel β-sheets surrounded by two α-helices, evidenced by the red circles in the Figures 1A and 1B, containing common sequences corresponding to 95–131 amino acid (a. a.) residues in spike RBD (Figure 1A) and 33–68 a.a. residues in the domain two of sFlt-1 protein (Figure 1B). So, similarity in the 3D structure of the two proteins in these particular regions leads to antibody cross-reactivity between the two proteins. Similar studies have been reported for spike RBD and platelets factor 4 (PF-4) interactions (Passariello M, et al, 2021).

```
4CKV_1|Chain 33-NITVTLKKFPLDTLIP---DGKRIIW DSRKGFIISNATY 68 a.a.
6W41_3|Chain 95 GQTGKIADYN--YKLPDDFTGCVIAW NSNNLDSKVGGNY' 131 a.a.
                .  * .: .:      :*    *  * * :*.:     ...*
```

Supplementary table 1: summarizes the similar amino acid residues between sFlt-1 (33 – 68 a.a) and spike RBD (95-131) which indicates positions with fully conserved residues or groups with strongly similar properties.

	* Indicates positions that have a single, fully conserved residue							: indicates conservation between groups of strongly similar properties - scoring > 0.5 in the Gonnet PAM 250 matrix					. indicates conservation between groups of weakly similar properties - scoring =< 0.5 in the Gonnet PAM 250 matrix.						
4CKV_1 (sFlt-1)	35 T	48 P	50 G	53 I	55 W	57 S	68 Y	38 L	41 F	47 I	56 D	59 K	33 N	37 T	40 K	58 R	65 N	66 A	67 T
6W41_3 SpikeRBD	97 T	108 P	113 G	116 I	118 W	120 S	131 Y	100 I	103 Y	107 L	119 N	122 N	95 G	99 K	102 D	121 N	128 G	129 G	130 N

Development of anti-spike monoclonal antibodies: Monoclonal antibodies (MAbs) were generated against rspike RBD protein, as described in the methods. The monoclonal antibody

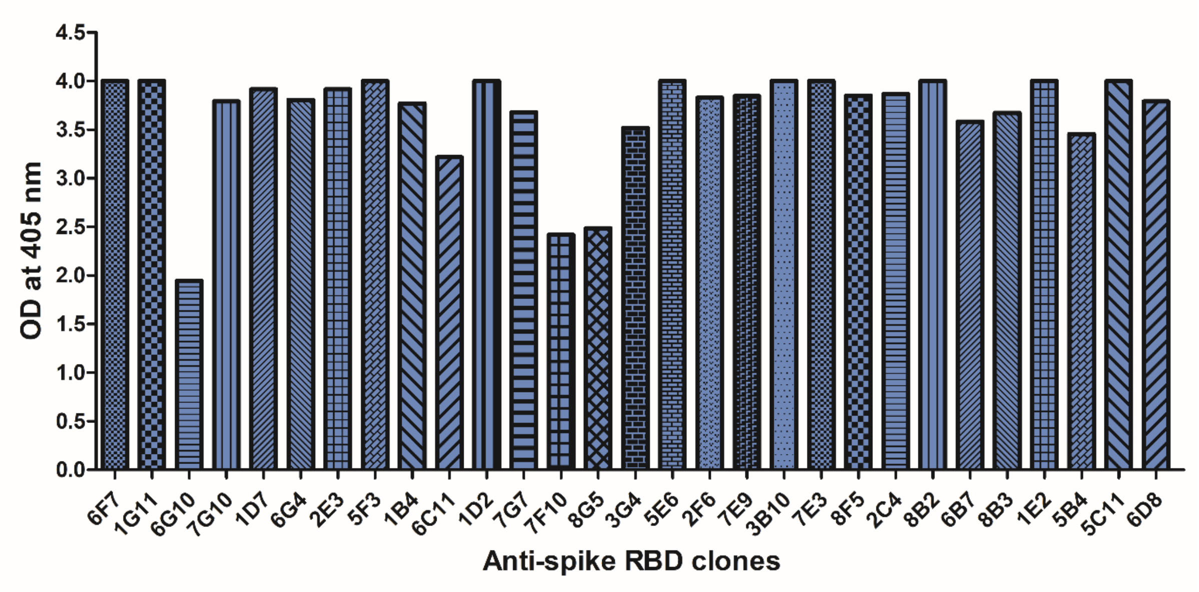

secreted by the anti-spike RBD clones showed varying reactivity to rspike RBD. The results show differential binding efficiencies to rspike RBD protein (figures S1 and S2).

Supplementary Figure 1: Absorbance (450 nm) of 29 positive anti-rspike RBD antibody-producing clones as observed by indirect ELISA.

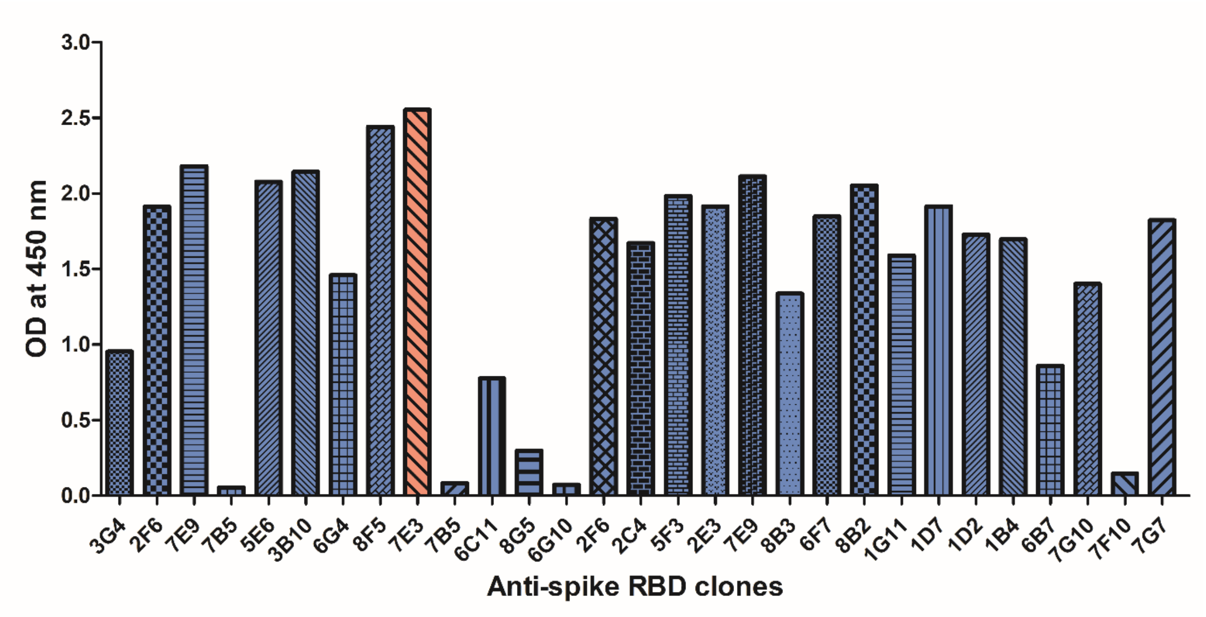

Supplementary Figure 2: Anti-rspike protein monoclones showing differential binding to rspike RBD protein. Red bar indicates the selected clone for rspike RBD and rsFlt-1 interaction recognition by indirect ELISA.

Confirmation of recombinant sFlt-1 and spike RBD expression and purification. We have expressed both spike RBD and sFlt-1 several times using our standardized procedure (Supplementary Figure 3). Every time we have confirmed the molecular weight of the expressed proteins both by silver staining and western blotting. sFlt-1 and spike RBD have been expressed by three independent experiments. These figures are only confirmatory assays for expressed recombinant proteins.

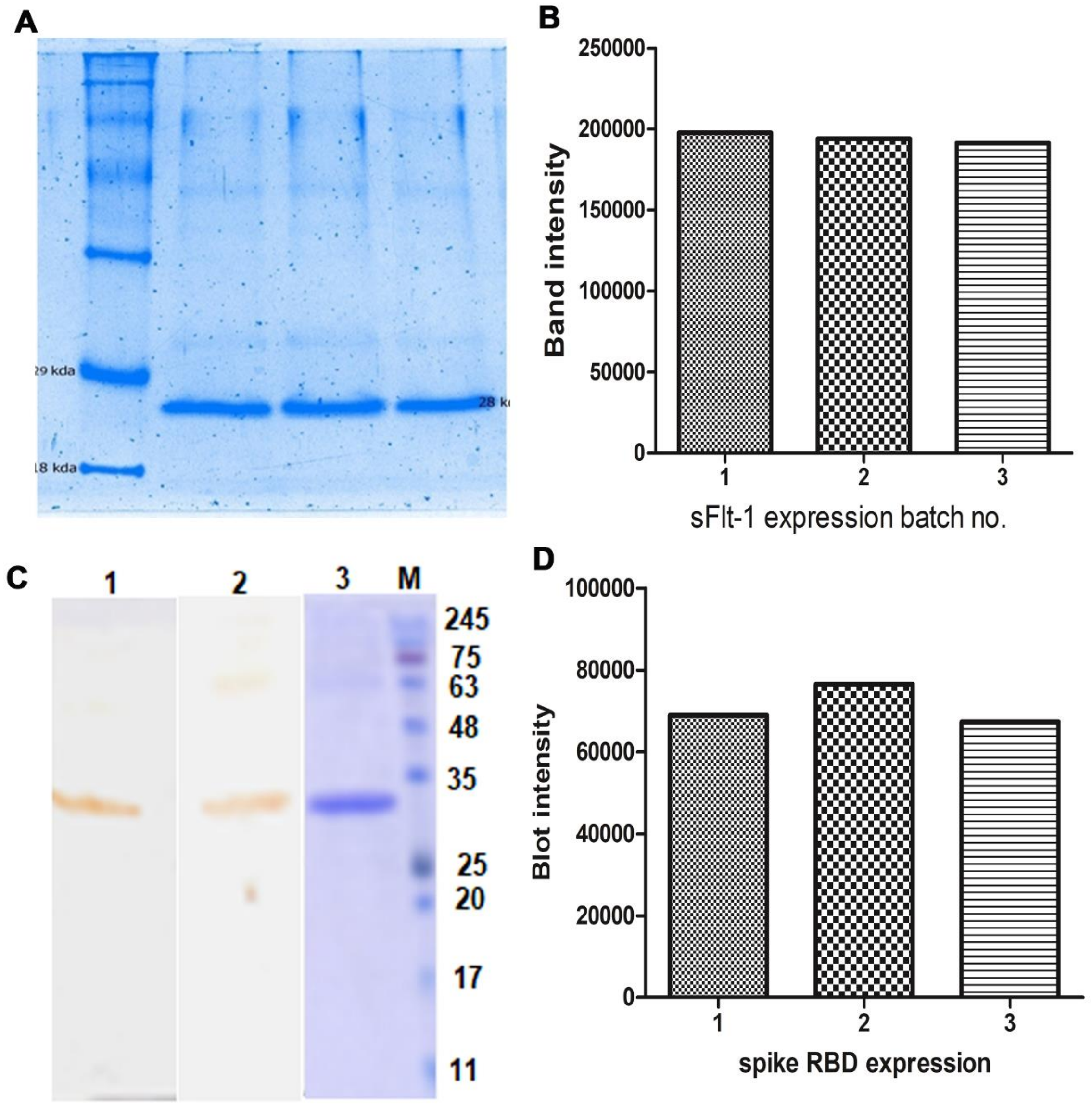

Supplementary figure 3: sFlt-1 and spike RBD were successfully expressed, purified and verified on SDS-PAGE and western blots: **(A)** sFlt-1 protein gel from three independent expressions batches stained with Coomassie brilliant blue reveals similar band intensity and purity. **(B)** representative graph of quantified bands using ImageJ software revealed similar bands in all the three independent expression batches (1,2,3 lane respectively). **(C)** Spike RBD protein SDS-PAGE gel and western blot result from 3 independent expression batches (1,2,3 lane respectively) revealed similar band intensity. In this experiment M: denotes the molecular weight marker. Both in-house produced anti-spike RBD antibody (Denovo Biolabs Pvt Ltd, India, lane 1) and commercially available rabbit anti-spike RBD antibody (Sigma Aldrich, SAB4200873, Lane 2), were used and reveals similar blot in all the independent batches.
